# The Rise of Apomixis in Natural Plant Populations

**DOI:** 10.3389/fpls.2019.00358

**Published:** 2019-04-02

**Authors:** Diego Hojsgaard, Elvira Hörandl

**Affiliations:** Department of Systematics, Biodiversity and Evolution of Plants (with Herbarium), Albrecht-von-Haller Institute for Plant Sciences, University of Göttingen, Göttingen, Germany

**Keywords:** apomictic crops, grass cultivars, polyploidy, reproductive assurance, sexuality, speciation, triploid bridge

## Abstract

Apomixis, the asexual reproduction via seed, has many potential applications for plant breeding by maintaining desirable genotypes over generations. Since most major crops do not express natural apomixis, it is useful to understand the origin and maintenance of apomixis in natural plant systems. Here, we review the state of knowledge on origin, establishment and maintenance of natural apomixis. Many studies suggest that hybridization, either on diploid or polyploid cytotypes, is a major trigger for the formation of unreduced female gametophytes, which represents the first step toward apomixis, and must be combined to parthenogenesis, the development of an unfertilized egg cell. Nevertheless, fertilization of endosperm is still needed for most apomictic plants. Coupling of these three steps appears to be a major constraint for shifts to natural apomixis. Adventitious embryony is another developmental pathway toward apomixis. Establishment of a newly arisen apomictic lineage is often fostered by side-effects of polyploidy. Polyploidy creates an immediate reproductive barrier against the diploid parental and progenitor populations; it can cause a breakdown of genetic self-incompatibility (SI) systems which is needed to establish self-fertility of pseudogamous apomictic lineages; and finally, polyploidy could indirectly help to establish an apomictic cytotype in a novel ecological niche by increasing adaptive potentials of the plants. This step may be followed by a phase of diversification and range expansion, mostly described as geographical parthenogenesis. The utilization of apomixis in crops must consider the potential risks of pollen transfer and introgression into sexual crop fields, which might be overcome by using pollen-sterile or cleistogamous variants. Another risk is the escape into natural vegetation and potential invasiveness of apomictic plants which needs careful management and consideration of ecological conditions.

## Introduction

Sexuality is well entrenched in all seed producing plants. Seeds are an integral part of diaspores that enhance plant dispersals and store all nutrients needed to start the new generation. Therefore, a wide variety of insects, animals and men found food resources in many plant seeds. The development of human societies is tightly linked to the domestication and improvement of crop species through artificial selection and genetic breeding (see e.g., Gupta, 2004). Both traditional and molecular plant breeding techniques are designed to modify and exploit sexuality to create new heterozygous seed varieties with desired allele combinations for high yield, resistance to different environmental stressors, or nutritionally enriched seeds (e.g., golden rice; see Dirks et al., 2009). However, the same sexual mechanisms that are manipulated to improve plant varieties (e.g., engineering meiosis by reverse breeding) are simultaneously the ones responsible for diminishing heterozygosity and segregating successful gene combinations (Lambing et al., 2017).

**FIGURE 1:**
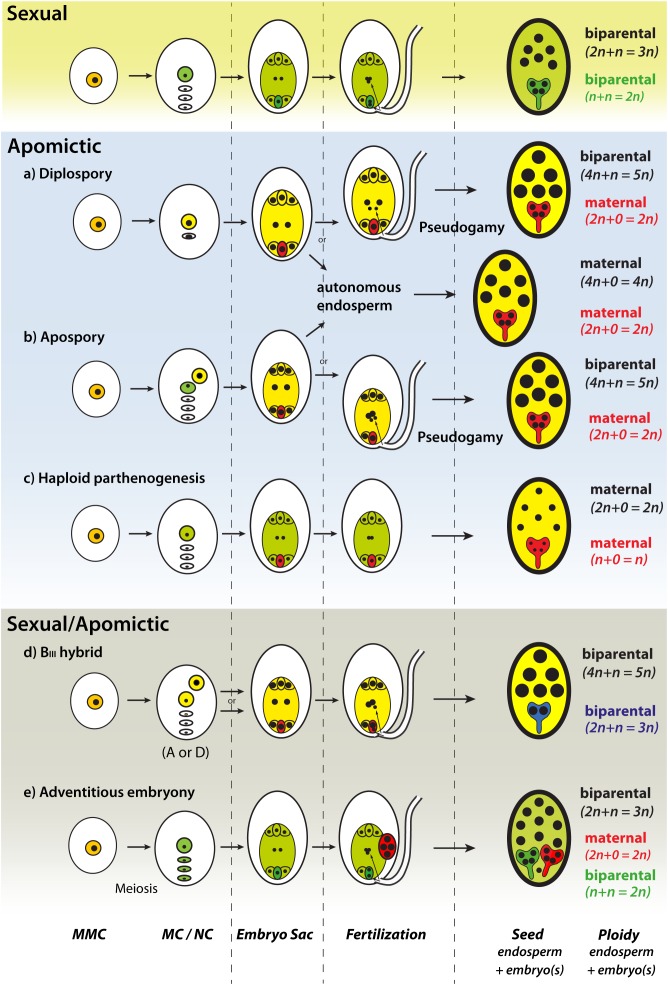
Main developmental pathways of natural apomixis in flowering plants (adapted from Hörandl, 2018). Meiotic developmental pathways **(c,e)** and biparental seed development **(d,e)** (green gametophytes, embryo, and endosperm tissues); apomictic developmental pathways **(a,b,d)** and maternal seed development **(a–c,e)** (yellow gametophytes and endosperm tissue, red egg-cell and embryo tissue); blue seed embryo is derived from a fertilized unreduced egg-cell. MMC, megaspore mother cell; MC, megaspore; NC, nucellus cell; B_III_ hybrid, offspring produced by fertilization of unreduced egg cells. Size of nuclei corresponds to relative ploidy level.

The formation of a seed involves a number of complex developmental steps, highly regulated and coordinated that still are not well understood (Bradford and Nonogaki, 2007). Sexual seed development is initiated by the process of double fertilization, which involves the fusion of reduced female and male gametes and leads to the development of the embryo and the endosperm (Figure 1). The hormone auxin has a crucial role during the initial development of seed structures and as a trigger of fertilization-independent seed development (Figueiredo and Kohler, 2018), a condition that occurs naturally in (apomictic) plants at low frequencies. Apomicts have evolved mechanisms that circumvent sexual pathways (Figure 1) by forming functional female gametophytes without meiosis (apomeiosis), developing embryos without fertilization (parthenogenesis), and a functional endosperm. Unreduced gametophytes can develop via two main developmental pathways: (1) two unreduced MCs are formed via restitutional meiosis or via mitotic division (diplospory); (2) a somatic, unreduced cell of the nucellus develops into an embryo sac (apospory). Although gamete fusion is a strict requirement for initiation of seed development in nature, apomictic plants can produce seeds through a single fertilization of the polar nuclei (pseudogamy) or without fertilization (autonomously) (Figure 1). Therefore, seed development without fertilization represents a trait of high economic relevance to exploit heterosis and preserve superior allele combinations (Koltunow and Grossniklaus, 2003). Synthetic clonal seed production had been exercised in both *Arabidopsis* and rice, aiming at introducing apomixis-like features into crops (Marimuthu et al., 2011; Mieulet et al., 2016). However, while cultivated crops are expected to be genetically uniform in a similar way to apomictic clone-mates, the introduction of apomixis in crop fields might bring new ecological threats derived from the biological advantages apomictic plants show compared to sexual ones (e.g., uniparental reproduction, unidirectional gene transfer; Hörandl, 2006). The escape of an apomixis gene into a wild relative may provide immediate invasive-like features to the recipient individual, but also other unintended (i.e., pleiotropic) benefits like increased fitness or pathogen resistance already observed in cases of crop x wild hybridizations (Chapman and Burke, 2006).

Before speculating about the biosafety and ecology of a potential apomictic crop, we can gain comparable valuable information from observations in natural apomictic plant populations. Apomicts exhibit a variety of developmental alternatives to bypass sexual pathways and produce clonal seeds (Figure 1). In single ovules, apomicts might use both sexual and apomictic seed development alternately (only one pathway proceed; Figure 1a,b) or even simultaneously (either both pathways proceed or are combined forming a B_III_; Figure 1d,e). Understanding the dynamics of apomixis in natural populations can provide useful information to know how an apomictic crop may behave in natural fields and visualize potential ecological threats. In recent years, the use of different technologies had enlarged our understanding of the genetic and developmental basis of apomixis in different plant species (Ozias-Akins and van Dijk, 2007; Conner et al., 2015; Hojsgaard and Hörandl, 2015) and has brought new light into initial steps and dynamics during the foundation and spread of new apomictic populations. Here we review main findings about the rise and dynamics of apomixis in natural plant populations.

## The Foundational Phase: the Emergence of an Apomictic Individual

Despite decades of research, it is still unclear how apomixis originates *de novo* in natural populations. Two main possibilities can be envisioned: either, seeds are dispersed from an apomictic source population, and the seedlings would find a new apomictic population; or, a spontaneous shift to apomixis happens in an otherwise sexually reproducing individual. The first case is difficult to trace in plants, as neither seed dispersal nor pollen dispersal can be easily documented in natural populations. Establishment of an apomictic newcomer in an otherwise sexual population is also hampered by minority effects (see below), and reduced fecundity (Hörandl and Temsch, 2009). Of course, the first scenario shifts the natural origin of apomixis just to another source population.

For the spontaneous *de novo* emergence of apomixis in natural populations, different hypotheses have been proposed. Traditionally, hybridization was regarded as a main trigger for emergence of apomixis (Ernst, 1918; Asker and Jerling, 1992; Carman, 1997). Evidence for hybrid origin of natural apomictic taxa is available in an increasing number of molecular studies (e.g., Koch et al., 2003; Paun et al., 2006; Lo et al., 2010; Beck et al., 2012; Šarhanová et al., 2017). The emergence of apomixis in hybrids has been confirmed even for diploid species. In fact, almost all natural diploid apomictic species in the genus *Boechera* are hybrids (Kantama et al., 2007; Aliyu et al., 2010; Beck et al., 2012). Synthetic diploid F_1_ hybrids in the *Ranunculus auricomus* complex of diploid, obligate sexual parental species showed spontaneous emergence of apospory in first hybrid generation (Hojsgaard et al., 2014a), and increased frequencies of apospory and first functional apomictic seeds in the diploid F_2_ (Barke et al., 2018). These studies also shed light on the open question why only few plant hybrid combinations would express spontaneous apomixis: hybridization appears to affect only one component of apomixis, i.e., the formation of an unreduced embryo sac from a diplosporous or aposporous initial cell. The other steps of apomictic seed formation, namely parthenogenesis and endosperm formation, are apparently not influenced by hybridization (Barke et al., 2018).

Other authors focused on polyploidization, following the observation that almost all natural apomictic plant populations are polyploids. Polyploidy could result in a “genomic shock” and genome-wide changes of gene expression (Koltunow and Grossniklaus, 2003). Carman (1997) developed the most comprehensive theory for polyploidization being the trigger for natural apomixis: climatic fluctuations during the Pleistocene would have caused range shifts and secondary contact hybridization of different ecotypes; the subsequent changes in timing of gene expression patterns in the cascade of megasporogenesis-megagametogenesis would be changed so that the megasporogenesis phase would be skipped, resulting in suppression of sexuality and expression of apomixis. In principle this could also happen after autopolyploidization in duplicated genes. Developmental and transcriptomic studies in fact revealed signs of asynchrony of gene expression in apomictic development (e.g., Polegri et al., 2010; Sharbel et al., 2010). In Paspalum notatum, artificial polyploidization led to the expression of apomixis in two synthetic autotetraploids while a third induced autopolyploid remained sexual (Quarin et al., 2001). Likewise, other autopolyploid *Paspalum* species, e.g., *P. plicatulum* and *P. simplex*, remained sexual after artificial polyploidization (Sartor et al., 2009).

In natural systems, the effects of polyploidy for the functionality of apomixis are not yet clear. A positive effect of autopolyploidization on establishing higher frequencies of apomictic seed formation has been observed in polyploidized *Paspalum rufum* (Delgado et al., 2014). Allele dosage effects of apospory or diplospory-specific genomic regions in polyploids on frequencies of apospory/diplospory have been observed in different model systems (Ozias-Akins and van Dijk, 2007). Dosage effects may further enhance development of unreduced embryo sac formation compared to meiotic reduced ones (Sharbel et al., 2010; Hojsgaard et al., 2013). A classical model by Nogler (1984) suggested that the apospory-controlling factors would have lethal effects in haploid gametes, thereby requiring diploid gametes for inheritance. However, this hypothesis was rejected by findings of Barke et al. (2018) that apospory can be inherited by haploid gametes in diploid *R. auricomus* hybrids.

**FIGURE 2:**
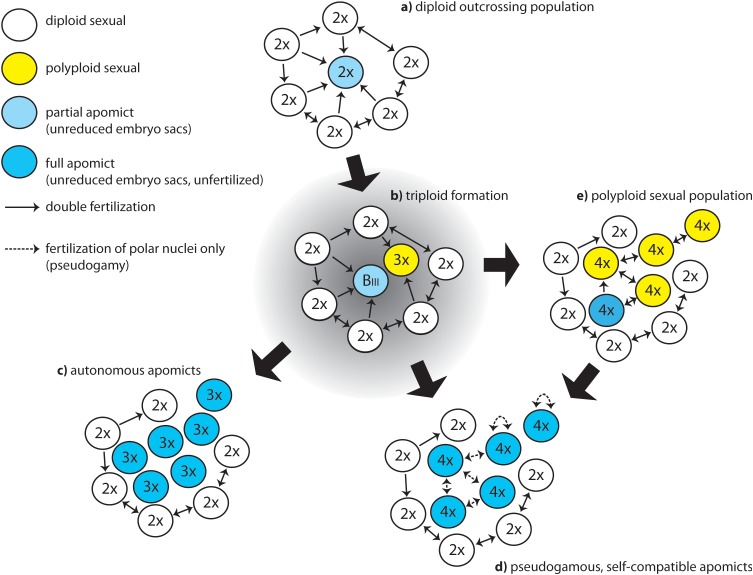
Hypothetical scheme of evolutionary pathways to apomixis in natural populations. **(a)** starting population; **(b)** emergence of apomixis elements and rise of first polyploid plants; **(c)** stabilization of functional, pollen-independent apomixis in triploids; **(d)** formation of even-ploid apomictic, pseudogamous polyploids; **(e)** formation of even polyploids and reversal to sexual reproduction.

Moreover, some model systems appear to express apomixis without any signs of hybridity or polyploidy. In *Paspalum*, many diploid species exhibit development of unreduced female gametophyte at low frequencies (reviewed in Ortiz et al., 2013), some of which seem to be able to have apomictic seed formation (Siena et al., 2008; Ortiz et al., 2013; Delgado et al., 2014, 2016). In the alpine species *Ranunculus kuepferi*, large scale FCSS screenings revealed spontaneous apomictic seed formation at low frequencies in otherwise sexual, diploid wild populations in the Alps (Schinkel et al., 2016). These diploid populations are not hybrids, they are geographically distant and isolated from each other and from apomictic tetraploids; no apparent dispersal or gene flow could be traced between them in population genetic studies (Cosendai et al., 2013). More detailed FCSS study further contradicted the hypothesis of a contagious origin of apomixis in diploids via pollination from tetraploid apomicts, but rather suggested a female triploid bridge of rare B_III_ hybrid formation, via female unreduced gametes produced by diploid plants (Schinkel et al., 2017; Figure 2). Among tetraploids of *R. kuepferi*, not even a single tetraploid obligate sexual population or individual could be found in the whole range of the species, which contradicts the idea that the shift to apomixis happened after polyploidization. Experimental studies rather suggested that cold shocks and frost treatments during development can increase frequencies of apomictic and also B_III_ seed formation in diploid *R. kuepferi* (Klatt et al., 2018). Although the frequencies of these events are low, they might be effective in evolutionary time periods.

What actually might trigger unreduced embryo sac formation under natural conditions? The appearance of apospory in otherwise sexual diploid plant species has been reported from *Paspalum* (reviewed by Ortiz et al., 2013), and in many Asteraceae genera that were otherwise not apomictic (Noyes, 2007). Aposporous initials act as surrogate cells for the meiotic products, or the spores, and ectopic, aposporous cell formation depends on production of a meiotic tetrad in *Hieracium* (Koltunow et al., 2011). This may not be the case in other apomictic systems like *Paspalum*, where the MMC often initiates abortion before entering the meiotic division (e.g., Hojsgaard et al., 2008). Cell-to-cell communication and/or direct contact between the emerging aposporous initial cell and the MC appears to take place before the former suppresses development of the latter (Schmidt et al., 2014, 2015; Juranic et al., 2018). Also, restitutional female meiosis, the process resulting in diplospory, is relatively widespread in plants. In *Taraxacum*, a model with first division restitution, the DIPLOSPORY (DIP) locus could be characterized and located on one NOR chromosome (Vijverberg et al., 2010; Vasut et al., 2014). Restitutional meiosis, however, can also be triggered by extreme temperatures and other environmental factors (De Storme and Geelen, 2013; Mirzaghaderi and Hörandl, 2016). In female development, unreduced MCs just have to develop into unreduced embryo sacs to produce unreduced female gametes. This capacity of unreduced gamete formation fits to the hypothesis discussed by Carman (1997); Hojsgaard et al. (2014b): that all angiosperms may have an inherent potential for shifting to apomixis.

Comparative transcriptomic studies on sexual and apomictic plants suggested that many stress-associated genes are differentially regulated in the premeiotic to gametophytic stage (Sharbel et al., 2010; Schmidt et al., 2014, 2015; Shah et al., 2016; Rodrigo et al., 2017). Prolonged photoperiods triggered increased sexual MC formation in facultative apomictic plants and resulted in reprogramming of secondary metabolite profiles (Klatt et al., 2016). Hence, natural apomeiosis has to be seen in the context of the physiological condition of the plant. This fits to a more general hypothesis that sexuality would have evolved and established early in eukaryote evolution as a DNA repair tool after oxidative stress conditions (Hörandl and Hadacek, 2013; Hörandl and Speijer, 2018). Fluctuating environmental conditions and stress response are hypothesized to be the major natural triggers for expressing the meiotic pathway. This aspect also sheds a new light on the putative role of polyploidy on expression of apomixis. Polyploids in general better regulate environmentally induced stress conditions, showing homeostatic maintenance of reproductive output under elevated abiotic stress, and therefore have a fitness advantage over diploids in climatically variable or extreme habitats (Schoenfelder and Fox, 2015). Hence, low stress conditions in polyploid reproductive tissues would stimulate the sexual meiotic pathway in archesporial cells to a lesser extent, thereby releasing the inherent potential of plants for apomeiosis (Hörandl and Hadacek, 2013).

In wild populations, the spontaneous appearance of the fully functional apomictic pathway is probably limited by the failure of connecting unreduced gamete formation to parthenogenesis and endosperm formation. Parthenogenesis itself is a process which again may occur spontaneously in natural populations. Again, the totipotency of plant cells allows for embryogenesis to start from different cell types, be it fertilized or unfertilized egg cells, somatic cells, or can be even induced in other tissues like microspores (Soriano et al., 2013). Somatic embryogenesis is in *Paspalum* associated with *SOMATIC EMBRYOGENESIS RECEPTOR-LIKE KINASE* (*SERK*) genes, and altered temporal and spatial expression of SERK gene copies appear to be associated with apomixis (Podio et al., 2014b). In apomictic *Paspalum* plants, cytosine-methylations inactivate genes that otherwise repress parthenogenesis (Podio et al., 2014a). In *Pennisetum* and in *Brachiaria*, the *ASGR-BABY BOOM*-like (*ASGR-BBML*) gene could be identified to control parthenogenesis (Conner et al., 2015; Worthington et al., 2019). In rice, ectopic expression of the *BABY BOOM1* (*BBM1*) gene results in parthenogenesis (Khanday et al., 2019). In *Hieracium* subg. *Pilosella*, LOSS OF APOMEIOSIS (LOA) and LOSS OF PARTHENOGENESIS (LOP) loci control apomixis, whereby gametophytic expression of LOP is required for both parthenogenesis and endosperm formation (Koltunow et al., 2011). The endosperm and parthenogenesis loci are linked but separate (Ogawa et al., 2013). In apomictic *Boechera*, genomic imprinting appears to be involved in the expression of parthenogenesis (Kirioukhova et al., 2018). In sexual *Boechera* species, paternal and maternal alleles are expressed for embryogenesis, while in parthenogenetic taxa, maternal expression of the *PHERES1* gene is drastically increased compared to sexual species. The changes in expression are probably due to altered DNA methylations. Reduced expression of Methyltransferase1 (MET1) and increased expression of Domains-arranged-Methyltransferases (DRM2) will cause cytosin-demethylations, resulting in the observed high expression levels of maternal *PHERES1* alleles (Kirioukhova et al., 2018).

In natural populations, haploid parthenogenesis has been reported as a rare event from many plants that is rarely successful (Asker and Jerling, 1992). Haploid embryos probably suffer too much from having just one chromosome set to establish a haploid progeny in natural environments. Polyhaploid progeny, however, was achieved in higher frequencies from 7x or 8x mother plants in *Hieracium* (Rosenbaumova et al., 2012). The authors suggested precocious embryogenesis controlled by gametophytes as a putative mechanism. Frequencies of polyhaploids in apomictic plant seed progenies are often very low (<5%) and they usually represent the smallest proportion of all developmental pathways (Bicknell et al., 2003; Kaushal et al., 2008; Krahulcova et al., 2011; Schinkel et al., 2017). Timing of pollination appears to be important for the expression of parthenogenesis. In many apomictic species, early pro-embryos had been observed at blooming (e.g., Cooper and Brink, 1949; Burson and Bennett, 1971; Hojsgaard et al., 2008), indicating accelerated parthenogenetic development in some ovules. Anticipated pollinations in facultative apomictic *P. notatum* were able to unlock the recalcitrant nature of unreduced egg cells to fertilization, increasing the formation of B_III_ progeny (Martinez et al., 1994). In a similar experiment but using different plant materials, Espinoza et al. (2002) could show experimentally in *P. notatum* that early pollination (before anthesis) and also late pollination (after anthesis) increased frequencies of apomictic offspring formation, while pollination during anthesis resulted in higher frequencies of sexual seeds. In natural plant populations, pollination during full anthesis is probably the most frequent “default” situation, because insects will be attracted by full floral displays, and wind-pollination is also most efficient in fully opened spikelets. Hence, pollination during anthesis in apomicts would maximize fertilization of reduced egg cells.

The overall data suggest that differential penetrance of parthenogenesis among ovules, carrying reduced and unreduced female gametophytes, might play a relevant role in creating the observed diversity of seed formation pathways. Under this context, a shift in timing of pollination in natural diploids, presenting low proportions of unreduced gametophytes, can significantly increase the relative success of unreduced gametophyte against reduced ones and favor the formation of asexual seeds. This may explain why the above-mentioned diploids of *P. rufum* (Delgado et al., 2014) or alpine *R. kuepferi* produced some fully apomictic seeds under wild conditions (Schinkel et al., 2016) and in experiments (Klatt et al., 2018). Accelerated flower development is a common feature of alpine plants, and a putative adaptation to short vegetation periods in alpine environments, especially in early flowering plants (Körner, 2003). Diploid *R. kuepferi* flowers directly after snow melting, a time when many insect pollinators are not yet available as a pollen vector. Hence we suppose that delayed pollination can easily happen under natural conditions, favoring occasional parthenogenetic development of unreduced egg cells. Further experimental work will be needed to understand the appearance of parthenogenesis under natural conditions.

Endosperm formation is under a different genetic or epigenetic control and is dependent on fertilization of polar nuclei (pseudogamy) in most natural apomicts. Therefore the endosperm and parthenogenesis loci are linked but separate. In Asteraceae, tissues in the ovule other than endosperm appear to provide sufficient nutrients for the embryo (Cooper and Brink, 1949). Likewise, plant families without endosperm formation in the seeds, i.e., Melastomataceae and Orchidaceae, can apparently express autonomous apomixis (Renner, 1989; Teppner, 1996; Zhang and Gao, 2018). Hence autonomous apomixis might evolve when the selective force for endosperm formation is weak. Pseudogamy, however, is predominant in most other families (Mogie, 1992) and is an important constraint for successful seed formation. Some species are sensitive to deviations from an 2 maternal : 1 paternal genome contribution in the endosperm while others are more tolerant (Talent and Dickinson, 2007). Precocious embryo development combined to late pollination would probably indirectly favor double fertilization of polar nuclei, as no receptive egg cells would be available when pollen tubes reach the micropyle. Both sperm nuclei would be directed to fertilize polar nuclei, which has, in *Polygonum* type embryo sacs, positive effects on endosperm development by maintaining 2 maternal : 1 paternal genome ratios (see above).

Taken together, the coupling of three developmental steps for functional apomictic seed formation is probably realized unfrequently in natural populations. The need of combining mutations for at least three developmental steps makes a mutagenic origin of apomixis in nature very unlikely, as each single component would be selected against (Van Dijk and Vijverberg, 2005). It rather seems that a coincidence of environmental conditions might alter developmental gene expression patterns, resulting in rare apomictic seed formation. Since apomeiosis avoids meiotic “resetting” of DNA methylation patterns (see Paszkowski and Grossniklaus, 2011), altered epigenetic states might be inherited in clonal seeds and may establish apomictic progeny.

Sporophytic apomixis, also called adventitious embryony includes embryogenesis out of somatic tissues of the nucellus or the integuments (Naumova, 1992). Apomictic embryos often develop from several initial cells in parallel or after sexual embryogenesis, resulting in more than one seedling within a seed (polyembryony). Although adventitious embryony is taxonomically the most widespread developmental pathway of apomixis (Hojsgaard et al., 2014b), the genetic control mechanisms are less well studied than in gametophytic apomixis. Because adventitious embryos arise without disturbing the sexual program, its genetic basis is expected to be less complex and a single mutation could initiate somatic embryogenesis. Genomic and transcriptomic analysis of *Citrus* species revealed 11 candidate loci associated to apomixis. An insertion at the promotor region of *CitRWP* is associated with polyembryony (Wang et al., 2017). Similar as in gametophytic apomixis, adventitious embryony often appears in polyploids and/or hybrids (Alves et al., 2016; Mendes et al., 2018), but also in diploids or paleopolyploids (Carman, 1997; Whitton et al., 2008).

## The Establishment Phase: the Formation of an Apomictic Population

During this phase, the uncoupled expression of apomixis developmental steps mentioned before is expected to be functional to the establishment of a polyploid apomictic population, required for the survival of the lineage. Uncoupled activation of apomeiosis and parthenogenesis in a diploid cytotype would drive an increase in ploidy and a shift in dosage that can help to stabilize the coordinated expression of apomixis elements and the formation of a number of polyploid individuals producing clonal seeds. In natural conditions, this mostly happens through a triploid intermediary that facilitates the formation of even polyploids, like in sexual systems. However, the presence of partial apomixis and uncoupled parthenogenesis can have different outcomes (Figure 2) and foster the establishment of new polyploid populations (Hojsgaard, 2018).

### Indirect Effects of Polyploidy

Polyploidization might have manifold indirect, positive effects on establishing apomictic individuals: first, polyploidy creates an immediate reproductive barrier against the diploid parental and progenitor population; second, polyploidy may cause a breakdown of genetic self-incompatibility (SI) systems which is needed to establish self-fertility of pseudogamous apomicts; and third, polyploidy could indirectly help to establish an apomictic cytotype in a novel ecological niche by changing the overall physiological features and adaptive potentials of the plants.

The interactions of cytotypes in populations with mixed cytotypes will likely lead to polyploidization of the offspring rather than increasing frequencies of occasional diploid apomictic individuals in a population. The following process can be envisioned: the appearance of a diploid apomeiotic individual within an otherwise diploid sexual, self-incompatible population will initially lead to a minority cytotype disadvantage (Levin, 1975), because mostly haploid pollen from the majority of surrounding sexual plants will be transferred to its stigmas (Figure 2a). The apomictic, diploid pioneer-producing unreduced embryo sacs will probably be mostly cross-fertilized and produce triploid B_III_ hybrid offspring, which means that hardly any diploid apomictic progeny can be formed (Figure 2b). In natural diploid populations, identification of apomictic progeny is difficult but feasible using the appropriate molecular approaches (e.g., Siena et al., 2008) or flow cytometric seed screenings (Schinkel et al., 2016). The experimental evidence support the mentioned idea of constraints to the formation of diploid apomictic progeny in nature (Siena et al., 2008; Hojsgaard et al., 2014a; Barke et al., 2018). An exceptional case is represented by apomictic diploids from *Boechera*. Different species within *Boechera* show a complex evolutionary history of hybridization and polyploidy, in which -besides the occurrence of apomictic triploids- diploid cytotypes can be sexual or apomictic, the latter being able to produce apomictic seeds recurrently (Aliyu et al., 2010). For details about the possible origin of the patterns of reproductive mode and ploidy variation observed across *Boechera* see Lovell et al. (2013). Once the mentioned B_III_ triploids are produced, apomeiosis may be more successful for female unreduced gamete formation as it circumvents meiosis dysfunction and the formation of aneuploid gametes. Microsporogenesis and pollen formation, however, will mostly fail producing an array of genetically and chromosomally unbalanced gametes rendering fertilizations unsuccessful, as observed in sexual triploids (e.g., Duszynska et al., 2013). Only parthenogenetic eutriploid embryos would develop, further skipping the molecular consequences of unbalanced genes and chromosomes observed in aneuploid embryos (Birchler and Veitia, 2012). When pollen is not essential for endosperm formation, as it is the case in most Asteraceae, then a triploid, pollen-sterile, highly obligate apomictic lineage would rapidly become established by selection against the sexual pathway (Figure 2c). This scenario is confirmed by the occurrence of different natural populations of triploid apomicts showing autonomous endosperm development in *Erigeron* (Noyes and Rieseberg, 2000), *Hieracium* (Bicknell et al., 2000), *Taraxacum* (Tas and van Dijk, 1999), and by a mathematical model for origins of 3x *Taraxacum* clones (Muralidhar and Haig, 2017). Recurrent formation of novel 3x dandelion clones can happen in mixed sexual/apomictic populations (Martonfiova, 2015). In pseudogamous diploid apomicts, triploid B_III_ cytotypes would probably not readily establish a population unless requirements for parental genomic contributions are relaxed, because pollen formation will be heavily disturbed in triploids and hamper proper endosperm formation. However, fertilization of unreduced 3x egg cells with well-developed haploid pollen from surrounding diploid sexuals can result in tetraploid plants in the next generation, as experimentally observed in most apomicts (Martínez et al., 2007; Hojsgaard et al., 2014a). In tetraploids, meiosis and pollen production is expected to be more stable, and diploid pollen will be available for pseudogamy. When the capacity for apomeiosis was inherited from the triploid mother, and coupling to parthenogenesis is successful, an apomictic tetraploid offspring could originate via a female triploid bridge (Figure 2d), as observed in *R. kuepferi* (Schinkel et al., 2017), *P. simplex* (Urbani et al., 2002) and likely in all apomictic systems where occasional triploids had been recorded in natural populations. Alternatively, if during the phase of establishment of the new population, apomixis cannot be stabilized in the new tetraploids but instead meiosis is re-installed and coupled to syngamy (Figure 2e), then sexual polyploidization could be the consequence of this transient B_III_ hybrid (Hojsgaard, 2018).

In some apomictic systems, sexual cytotypes are polyploid and no evidence of occurrence of diploid cytotypes is found in nature. Here we might consider two alternative explanations. In one case, sexual diploids could first undergo sexual polyploidization via unreduced male gametes fertilizing reduced egg cells (male triploid bridge) (De Storme and Geelen, 2013), and then become extinct. The second possibility is that a polyploid apomict in an agamic complex reverts to sexuality, while diploid sexuals become extinct, and then it produces higher ploidy apomictic cytotypes by repeating the cycle. In both cases, among sexual tetraploids, a similar mechanism of rare apomictic seed formation as in diploids might start from predominantly sexual tetraploid populations, like in *Potentilla puberula*, where just single individuals showed some apomixis, whereas predominant apomixis occurred in cytotypes with higher ploidies (5x to 8x cytotypes; Dobes et al., 2013). Once the high polyploid apomictic lineage is established, heteroploid cross-fertilizations will rather negatively influence fertility of the lower-ploid sexuals, but not the fitness of the higher ploid apomictic plants (Dobes et al., 2018). A similar cytotype distribution and reproductive features was observed, for example, in *H. pilosella* (=*Pilosella officinarum*; Mráz et al., 2008), in *Paspalum durifolium* or in *P. ionanthum* (Ortiz et al., 2013). Seed abortion in sexuals after heteroploid cross-fertilization versus high female fitness after homoploid crosses, but also induced selfing (Mentor effects), can contribute to maintenance of diploid sexual populations (Hörandl and Temsch, 2009).

An important side-effect of polyploidization is breakdown of SC systems (SI), resulting in self-fertility, as it is also well known from sexual polyploids (Comai, 2005; Hörandl, 2010). SI systems act in the stigma and in the style, and have a genetic control independent from embryo sac development by S-alleles (de Nettancourt, 2001). Nevertheless, an important selective mechanism can help to establish polyploid, pseudogamous, self-compatible (SC) clonal lineages: a self-incompatible apomictic plant can neither use its own pollen, nor the pollen of genetically identical clone-mates around, because the S allele configuration will be the same in surrounding clone-mates. In contrast, an apomictic self-compatible pioneer plant can not only use self-pollen for pseudogamy, it can also use pollen of surrounding clone-mates with identical genotypes for seed production (Hörandl, 2010). In this way, the newly formed self-compatible polyploid clone becomes completely independent from pollen of surrounding sexual progenitors (Figure 2d). By using self-pollen, an appropriate endosperm balance can also be more easily achieved. Self-fertility with pseudogamy further avoids the negative effects of sexual selfing, namely loss of heterozygosity and inbreeding depression (Hörandl, 2010). Self-fertility is further beneficial for founding new populations by single or few founders, even after long distance dispersal of seeds (Baker, 1967; Hörandl, 2006; Cosendai et al., 2013).

Taken together, a couple of internal and external factors have to coincide to combine the different steps of apomixis. Under natural conditions, functional apomictic seed formation in diploids probably requires certain altered ecological conditions, and successful polyploidization for establishment. The rarity of events, which have to be combined, may also cause the low actual frequencies of natural apomixis in angiosperms (see Hojsgaard et al., 2014b for a recent review).

## The Diversification Phase: Range Expansion and Speciation of Apomicts

Once an apomictic polyploid population is established, its survival will depend upon the neopolyploids’ capacity to either outcompete parental diploids, or move into another habitat. The occupation of a novel ecological niche is in many cases a side-effect of polyploidy. As discussed above, polyploidy *per se* alters many physiological and cellular features, which may be advantageous in a novel environment. This aspect has attracted much attention in the past, and some authors have seen the ecological potential of polyploidy as the main factor for the wide distribution of some apomicts (Bierzychudek, 1985). Niche shifts of polyploids compared to diploids have been documented in allopolyploid *Crataegus* (Coughlan et al., 2017), but also in the autopolyploids *R. kuepferi* (Kirchheimer et al., 2016, 2018) and *Paspalum intermedium* (Karunarathne et al., 2018). These non-hybrid apomicts do not show a pronounced genetic diversification, but nevertheless managed to occupy habitats outside the ecological range of the diploids. The evidence indicates that polyploid apomicts behave as generalists and are less competitive than specialist diploids in their native range, but they are more competitive in the peripheral areas of parental diploids (Karunarathne et al., 2018), a condition that may prelude ecological differentiation between cytotypes. Together with inherent biological features of apomixis (i.e., reproductive assurance and clonality), ecological niche shift is another important factor for geographical range expansions and diversification of apomicts. If the new apomictic polyploids cannot adapt to novel environmental conditions, their evolutionary potential would likely be restricted. Mau et al. (2015) found widespread niche conservatism between sexual and apomicts, and found that ploidy is a stronger driver for niche divergence compared to reproductive mode in diploid-triploid cytotypes of *Boechera*, but substantial variation in directions of niche differentiation was found among species. Thus, according to Mau et al. (2015), homoploid apomicts in *Boechera* are trapped in the ecological niches of their sexual ancestors.

### Reproductive Assurance and Clonality

Self-fertility and apomixis enable plants to form new populations starting with a single individual (Baker, 1955). Thus, plants benefit twice from apomixis. On the one hand, apomixis allows for founder events and spread of species’ populations after seed dispersal. On the other hand, apomixis creates clonal offspring and hence it multiplies genotypes, and it is likely that the fittest ones locally will be established by differential seed sets and better competing aptitudes. This double advantage provides plants with better colonizing abilities and both have a relevant impact on genetic variation at population level and in the biogeographic distribution of cytotypes. These combined features contribute to observed patterns of geographical parthenogenesis (e.g., Kearney, 2005; Hörandl, 2006).

### Geographical Parthenogenesis

Asexual animals and plants often have larger distribution areas than their sexual relatives (Kearney, 2005). In plants, the strong co-occurrence of apomixis and polyploidy made it difficult to entangle their effects on colonization patterns (Bierzychudek, 1985; Hörandl, 2006). In the exceptional case of the *Boechera holboellii* complex, Mau et al. (2015) found stronger evidence for ploidy driven ecological-niche divergence rather than for reproductive systems. Despite the fact that this observation contradicts general and well-supported patterns of geographic parthenogenesis, all other studies suggest apomixis might speed up dispersal and facilitate range expansions in those polyploids. In the alpine species *R. kuepferi*, diploid cytotypes remained in their refugial area in the southwestern Alps, while tetraploids colonized the whole Alps, the Apennines, and Corsica (Cosendai and Hörandl, 2010). Only tetraploids managed to adapt higher elevations and a colder climatic niche (Kirchheimer et al., 2016; Schinkel et al., 2017). A simulation study of recolonization of the Alps revealed strong combinational effects of niche differentiation and mode of reproduction for tetraploids (Kirchheimer et al., 2018). Similarly, in prairies and grasslands that were not associated to ice-sheet covers during the last glaciation, species also depict patterns of unequal geographic distribution between sexual and apomictic cytotypes. In the grass species *P. intermedium*, diploid cytotypes are less geographically expanded than tetraploid cytotypes and located in northern, climatically milder areas within the distribution of the species in South America (Karunarathne et al., 2018). Tetraploids occupied southern areas by better coping with less productive and harsher environmental variation (Karunarathne et al., 2018). Thus, in most plant systems, apomixis promotes range expansions by exploiting the advantages of clonality and polyploidy.

### Population Differentiation and Speciation

As apomixis freezes genetic variation and reduces genotype variability, populations are expected to evolve independently by reduced gene flow. With time they may evolve into populations holding gene pools that are differentiated enough to avoid hybridization via pre- and postzygotic barriers, and geographically distant (isolated) populations may become subspecies or new species (e.g., Arrigo and Barker, 2012). In fact, morphological differentiation is observed within apomictic complexes, whereby facultative apomixis in hybrid species can promote (slow) divergent selection and formation of microspecies (i.e., an apomictic lineage with particular morphology and genetically homogeneous) (e.g., Burgess et al., 2014), causing severe problems in taxonomy. For a detailed analysis on how to treat apomictic taxa and which species concepts to refer to Haveman (2013), Majeský et al. (2017), Hörandl (2018). An alternative would be to have a reversal to sexuality in one of those widespread populations (Hörandl and Hojsgaard, 2012). A shift back to sexual reproduction in a distant population from the parental sexual species of the polyploid apomicts would also allow for independent evolution, the accumulation of genetic and morphological changes and the acquisition pre- and postzygotic barriers to gene flow (Hojsgaard and Hörandl, 2015).

## Natural VS. Cultivated Apomictic Plant Systems

Apomictic plant systems produce clones from seeds, a trait that offers enormous potential for the development of cultivars specifically suited to livestock pastures. Natural apomictic populations are dynamic. Evidence suggests that they are often founded by a single individual that multiplies that genotype to establish a small local population. Along time, likely as a response to local edaphic conditions and environmental variation, the population acquires variation mainly due to residual sexuality and spontaneous mutations. Thus, natural apomictic populations are genotypically diverse (e.g., Daurelio et al., 2004; Paun et al., 2006).

Cultivated crops consist of genetically highly uniform individuals, usually derived from crosses between inbreed lines or highly selected heterozygous plant materials. In this sense, apomictic natural populations with genetically uniform clone-mates and pedigree cultivars of apomictic forage grasses are qualitatively similar, and to some extend comparable to cultivated crop fields with variable levels of heterozygosity and a contrasting type of reproduction involve in the formation of the next generation of seeds. Plant reproductive processes, flowering time, formation of floral organs, pollen viability, etc., are strongly influenced by climatic conditions and can affect seed yields and crop performance (Hampton et al., 2016). Climatic factors govern crop growth and development and are subject to spatial changes in both direction and magnitude, making necessary fine scale analyses to discern the differential spatial responses of crops to climate variability and their impacts on crop yields (Kukal and Irmak, 2018). Therefore, genetically pure sexual crops and apomictic cultivars are expected to have alike responses to variable environmental conditions which might help us understand the short-term effects of ecological and climatic changes on seed production and crop yields, and estimate potential climate-influenced crop yield gain/loss expected from the transfer of apomixis to main crops. Since most apomicts are facultative, in both natural clonal lineages as in commercial forage cultivars, the formation of low proportions of recombinant progeny is expected to increase genetic heterogeneity and inbreeding which may decrease forage productivity. Therefore, at least in forage breeding, a proper reproductive characterization of the selected material using multiple experimental approaches can benefit both breeding programs, by knowing potential rates of hybridization and trait introgression, and field management strategies by estimating proportions of non-maternal offspring expected per generation (Hojsgaard et al., 2016).

Most natural apomicts maintain the pollen function for pseudogamy. For apomictic crops, this means that they could act as pollen donors and introgress adjacent sexual crop stands (van Dijk and van Damme, 2000). To avoid introgression of apomictic variants into sexual ones, apomictic crops would have to be pollen-sterile. However, natural systems show us that the pollen function can be only abandoned with autonomous apomixis, which occurs mostly in plants with or without a weak endosperm formation (see above). Hence, breeding strategies for pollen-sterile apomictic cultivars may be most useful for forage plants where the major interest of the farmer is in vegetative growth. But for crops plants which are mostly cultivated for their seed yield, the need of pseudogamy for proper endosperm formation requires functional pollen. Breeding strategies aiming at enforced autogamy (e.g., within cleistogamous spikelets or flowers) in crop variants may help to overcome this problem.

Most domesticated crops are highly dependent on use of high inputs (e.g., fertilizers and herbicides), and therefore an escape from cultivation is less likely than an introgression event to a wild relative species (e.g., Arnaud et al., 2003; Uwimana et al., 2012). In contrast, apomictic forage crops are less dependent on inputs and can both introgress a wild relative and escape from cultivation. The same features that make apomictic crops suitable for development of cultivars also make them better invaders, instigating a potential threat to biodiversity and environmental risk. In Latin America, for example, superior *Brachiaria* grasses for livestock production have been widely adopted covering approximately 25 million hectares^1^, and many areas of Brazil have been recorded as invasive (Almeida-Neto et al., 2010; under the name *Urochloa* spp. in Zenni and Ziller, 2011). Similar cases are recognized worldwide but little is known about the adverse impacts of such invasions on biodiversity. A comparative study on sexual and apomictic invasive plants showed that the latter have similar abilities for ecological niche shifts and establishment in novel, invaded areas like sexual species (Dellinger et al., 2016). A few studied cases show apomictic grasses dominating invaded habitats and displacing native grasslands (Marshall et al., 2012; Dennhardt et al., 2016). Thus, a better understanding surrounding the origin and dynamics of natural apomictic populations, as well as the variation in the expression of residual sexuality and other sources of genetic variation, can help identify and target effective management actions for apomictic crops, which might currently contribute to the sustainable intensification of forage-based systems.

## Conclusion and Outlook for Future Studies

Apomixis is a complex, developmental trait expected to have an enormous impact in plant breeding if introduced into main crops, both shortening the time required to develop a new variety and to increase revenues. Currently, apomixis is exploited for the creation of forage cultivars but reports documenting, e.g., lack of genetic homogeneity and genetic erosion in those cultivars are not available. Although eclipsed for a long time, the mechanisms responsible for the rise and dynamics of apomixis in natural plant populations, the potential outcomes of apomixis in natural systems and its role in plant evolution start being disentangled. The knowledge about the genetic and ecological factors governing developmental interactions between meiotic and apomictic pathways, as well as the population dynamics at local and regional scales can not only help us to decipher the strategies plants use to respond and adapt to the environment, but it also provides valuable information to use on apomictic crop management and production practices.

## Data Availability

No original datasets were produced for this article.

## Author Contributions

All authors listed have made a substantial, direct and intellectual contribution to the work, and approved it for publication.

## Conflict of Interest Statement

The authors declare that the research was conducted in the absence of any commercial or financial relationships that could be construed as a potential conflict of interest.

## Footnotes

1 https://ciat.cgiar.org/

## Abbreviations

B_III_ hybrid: offspring produced by fertilization of unreduced egg cells
CitRWP: a RWP-RK domain–containing protein found in citrus
FCSS: flow cytometric seed screen
MC: megaspore
MMC: megaspore mother cell
NC: nucellus cell
PHERES1: a transcription factor encoded by a MADS-box gene
SC: self-compatibility
